# Development and clinical applications of cancer immunotherapy against PD-1 signaling pathway

**DOI:** 10.1186/s12929-019-0588-8

**Published:** 2019-12-05

**Authors:** Grace Wakabayashi, Yu-Ching Lee, Frank Luh, Chun-Nan Kuo, Wei-Chao Chang, Yun Yen

**Affiliations:** 1 Taipei Medical University, 250 Wu-Hsing Street, Taipei, Taiwan 110; 2 Center for Cancer Transnational Research, Taipei Medical University, 250 Wu-Hsing Street, Taipei, Taiwan 110; 3 Sino-American Cancer Foundation, 668 Arrow Grand Circle, Suite 101, Covina, California, 91722 USA; 4 Department of Clinical Pharmacy, School of Pharmacy, Taipei Medical University; Department of Pharmacy, Integrative Therapy Center for Gastroenterologic Cancers, Wan Fang Hospital; Taipei Medical University, 250 Wu-Hsing Street, Taipei, Taiwan 110; 5 PhD Program for Cancer Biology and Drug Discovery, Taipei Medical University, 250 Wu-Hsing Street, Taipei, Taiwan 110

**Keywords:** Checkpoint inhibitor, Cancer immunotherapy, PD-1 PD-L1 signaling

## Abstract

Dramatic advances in immune therapy have emerged as a promising strategy in cancer therapeutics. In addition to chemotherapy and radiotherapy, inhibitors targeting immune-checkpoint molecules such as cytotoxic T-lymphocyte antigen-4 (CTLA-4), programmed cell death receptor-1 (PD-1) and its ligand (PD-L1) demonstrate impressive clinical benefits in clinical trials. In this review, we present background information about therapies involving PD-1/PD-L1 blockade and provide an overview of current clinical trials. Furthermore, we present recent advances involving predictive biomarkers associated with positive therapeutic outcomes in cancer immunotherapy.

## Background

In 1992, Honjo et al. from Kyoto University discovered PD-1, a 228 amino acid transmembrane protein expressed in T-cells linked to apoptosis pathway [1]. Subsequent PD-1 mouse studies revealed the immunosuppressive effect of PD-1 knockout. PD-1 deficiency in BALB/c mice cause a variety of autoimmune diseases including dilated cardiomyopathy and gastritis [2, 3]. PD-1 is expressed in dendritic cells (DCs), B cells and activated T cells [4]. The ligands of PD-1/PD-L1 (B7-H1, CD274) and PD-L2 (B7-DC, CD273) were identified in 2000 and 2001, respectively [5–7]. PD-L1 is widely expressed in T cells and endothelial cells and is over expressed in different types of tumor cells. Upon PD-L1 binding to PD-1, T-cell receptor (TCR) signaling is inactivated following SHP2 dephosphorylation. This signaling inactivation suppresses T cell proliferation, cytokine release, and cytotoxic activity [8]. Experiments in tumor animal models indicate that inhibition of PD-L1 and PD-1 can block tumorigenesis and tumor metastasis via PD-1 mediated T cell activation, a key step for cancer immune therapy [9]. In 2006, Nivolumab, a humanized PD-1 mAb, was developed for phase I clinical trial and was eventually approved by the U.S. Food and Drug Administration (FDA) for patients with malignant melanoma in 2015. Currently, there are six FDA-approved PD-1/PD-L1 pathway inhibitors for cancer therapy: nivolumab, pembrolizumab, atezolizumab, durvalumab, cemiplimab and avelumab (Table 1).

**Table 1:** US FDA approved PD-1/PD-L1 inhibitors

Target	Molecule	Approved indications	Company	Commercial name
PD-1	Nivolumab (BMS-936558, MDX1106, ONO4538)	CRC (MSI high), HNSCC, HCC, Hodgkin lymphoma, melanoma, NSCLC, RCC, SCLC, UC	Bristol-Meyers Squibb/Ono	Opdivo
	Pembrolizumab (MK-3475, Lambrolizumab)	Cervical cancer, CRC, endometrial cancer, esophageal cancer, gastric cancer, HNSCC, HCC, Hodgkin lymphoma, melanoma, Merkel cell carcinoma, MSI high cancer, NSCLC, PMBCL, RCC, SCLC, UC	Merck	Keytruda
	Cemiplimab	Cutaneous squamous cell carcinoma	Sanofi	Libtayo
PD-L1	Atezolizumab (MPDL3280A)	Breast cancer, NSCLC, SCLC, UC	Roche/Genentech	Tecentriq
	Durvalumab (MEDI4736)	NSCLC, UC	MedImmune/AstraZeneca/ Celgene	Imfinzi
	Avelumab (MSB0010718C)	Merkel cell carcinoma, RCC, UC	Merck Serono/Pfizer	Bavencio

*CRC* Colorectal cancer, *HCC* Hepatocellular carcinoma, *HNSCC* Head and neck squamous cell carcinoma, *MSI* Microsatellite instability, *NSCLC* Non-small cell lung cancer, *PMBCL* Primary mediastinal large B cell lymphoma, *RCC* Renal cell carcinoma, *SCLC* Small cell lung cancer, *UC* Urothelial carcinoma

### Overview of PD-1/PD-L1 and other immune blockades in clinical trials

Immuno-oncology has proven to be a field with untapped potential in the fight against cancer. Many clinical trials are currently testing different ways to program the body’s immune system to target and eliminate tumors. Originally, studies on immune-checkpoint inhibitors (ICIs) focused on certain types of cancers but recent advances in science and research have allowed ICIs to target broader cancer types. Among the most well studied ICIs are monoclonal antibody therapies against PD-1 and PD-L1.

New insight on the interaction between the immune system and tumor growth has identified the PD-1/PD-L1 ligand pathway to be a key player in evading host immune response. By blocking this pathway, checkpoint inhibitors can reprogram the immune system to recognize tumor cells and ultimately destroy them. PD-1/PD-L1 inhibitors have been FDA approved for a wide variety of cancers (Table 1). The majority of published clinical trials have explored use of PD-1/PD-L1 inhibitors in patients diagnosed with melanoma, kidney cancer, head and neck, and non-small cell lung cancer (NSCLC) (Table 2). This review will focus on selected trials involving these cancers.

**Table 2:** Selected clinical trials of PD-1/PD-L1 immunotherapies according to cancer type

Trial	Subject	Study vs. comparison	Result	Reference	FDA approval outcome
Melanoma					
Keynote 006	No prior immunotherapy, any PD-L1 level	P 10 mg/kg vs. I	OS: 32.7 vs. 15.9 months PFS: 8.4 vs. 3.4 months	Schachter et al. 2017	FDA approved pembrolizumab for first-line treatment in advanced melanoma
Keynote 002	With prior ipilimumab, any PD-L1 level	P 2 mg/kg vs. P 10 mg/kg vs C	PFS: 34% vs. 38% vs. 16%	Ribas et al. 2015	FDA approved pembrolizumab for second-line treatment in advanced melanoma
CheckMate 066	Previous untreated, any PD-L1 level	N 3 mg/kg vs. dacarbazine	OS: 37.5vs. 11.2 months PFS: 5.1 vs. 2.2 months	Ascierto et al. 2019	FDA approved Opdivo for treatment of BRAF V600 wild-type unresectable or metastatic melanoma
CheckMate 037	With prior ipilimumab, any PD-L1 level	N 3 mg/kg vs. C	OS: 16 vs. 14 months PFS: 3.1 vs. 3.7 months ORR: 27% vs. 10%	Larkin et al. 2018	FDA approved Opdivo for unresectable or metastatic melanoma following treatment with ipilimumab or BRAF inhibitor
CheckMate 067	Previous untreated, any PD-L1 level	N 3 mg/kg + I 3 mg/kg vs. N 3 mg/kg vs. I 3 mg/kg	OS: not reached vs. 37.6 vs. 19.9 months PFS: 11.5 vs. 6.9 vs. 2.9 months	Larkin et al. 2015	FDA approved nivolumab in combination with ipilimumab for treatment of BRAFV600 wild-type and BRAF V600 mutation positive unresectable or metastatic melanoma
CheckMate 511	Previous untreated, any PD-L1 level	N 3 mg/kg + I 1 mg/kg vs. N 1 mg/kg + I 3 mg/kg	PFS: 9.9 vs. 8.9 months ORR: 45.6% vs. 50.6% Grade 3 to 5 AEs: 34% vs. 48%	Lebbe et al. 2019	
Renal Cell Carcinoma					
CheckMate 025	With prior treatment, any PD-L1 level	N 3 mg/kg vs. everolimus	OS: 25 vs. 19.6 months ORR: 22% vs. 4%	Escudier et al. 2017	FDA approved nivolumab for treatment of advanced renal cell carcinoma with no prior anti-angiogenic therapy
CheckMate 214	Previous untreated intermediate to poor risk, any PD-L1 level	N 3 mg/kg + I 1 mg/kg vs. sunitinib	OS: not reached vs. 26.6 months PFS: 8.2 vs. 8.3 months ORR: 42% vs. 29%	Motzer et al. 2019; Escudier et al. 2017	FDA approved nivolumab and ipilimumab for treatment of intermediate or poor risk, previously untreated advanced renal cell carcinoma
Non-Small Cell Lung Cancer					
Keynote 024	Previous untreated, with TPS over 50%	P 200 mg vs. C	OS: 80.2% vs. 72.4% PFS: 10.3 vs. 6 months ORR: 44.8% vs. 27.8%	Reck et al. 2016	FDA approved pembrolizumab for treatment of metastatic NSCLC whose tumors have high PD-LA expression with no EGFR or ALK genomic tumor aberrations
Keynote 189	Previous untreated, any PD-L1 level	P 200 mg + C vs. C	OS: not reached vs. 11.3 months PFS: 8.8 vs. 4.9 months	Gandhi et al. 2018	FDA approved pembrolizumab in combination with pemetrexed and platinum chemotherapy for first line treatment of metastatic non squamous NSCLC with no EGFR or ALK genomic tumor aberrations
Keynote 010	With prior treatment, any PD-L1 level	P 2 mg/kg vs. P 10 mg/kg vs. docetaxel	Total population OS: 10.4 vs. 12.7 vs. 8.5 months PFS: 3.9 vs. 4.0 vs. 4.0 months TPS ≥ 50% OS: 14.9 vs. 17.3 vs. 8.2 months PFS: 5.0 vs. 5.2 vs. 4.1 months	Herbst et al. 2016	FDA approved pembrolizumab as second-line treatment for PD-L1 Positive non-small cell lung cancer
IMpower 150	Nonsquamous, previous untreated, any PD-L1 level	A 1200 mg + C + bevacizumab 15 mg/kg vs. C + bevacizumab	PFS: 8.3 vs. 6.8 months	Socinski et al., 2018	FDA approved atezolizumab in combination with bevacizumab, paclitaxel, and carboplatin for first line treatment of metastatic non-squamous non-small cell lung cancer with no EGFR or ALK genomic tumor aberrations
Head and Neck Cancer					
Keynote 040	With prior treatment, any PD-L1 level	P 200 mg vs. C	OS: 8.4 vs. 6.9 months	Cohen et al. 2018	FDA approved pembrolizumab for treatment of recurrent or metastatic squamous cell carcinoma of head and neck with disease progression on or after platinum-based therapy
CheckMate 141	With prior treatment, any PD-L1 level	N 3 mg/kg vs. C	OS7.5 vs. 5.1 months	Kiyota et al. 2017	FDA approved nivolumab for treatment of recurrent or metastatic squamous cell carcinoma of head and neck with disease progression on or after platinum-based therapy

*A* Atezolizumab, *AEs* Adverse events, *C* Chemotherapy, *D* Durvalumab, *I* Ipilimumab, *N*, Nivolumab, *ORR* Objective response rate, *OS* Overall survival, *P* Pembrolizumab, *PFS* Progression-free survival, *TPS* Tumor proportion score

Historically, PD-1/PD-L1 clinical trials have explored the efficacy of combination chemotherapies with checkpoint inhibitors and use of checkpoint inhibitors as monotherapy. KEYNOTE-006, − 002, CheckMate-066 and -037 studies showed PD-1 inhibitors are beneficial for patients with advanced melanoma [10–13]. The PD-1 inhibitors in these trials produced an overall survival (OS) ranging from 16 to 38 months versus the comparative treatment’s OS of 11.2–15.9 months [10, 11, 13]. In CheckMate-025 and -214, urologic cancers, such as metastatic renal cell cancer, reported better clinical outcomes when patients are treated with nivolumab either as monotherapy or combined with ipilimumab (CTLA-4 inhibitor), compared to target therapy alone [14–16]. The overall response rate (ORR) in CheckMate-025 and -214 favored nivolumab over other treatments (22–42% vs. 4–29%) [14, 16]. Head and neck squamous cell carcinoma (HNSCC) trials such as CheckMate-141 and KEYNOTE 040 proved checkpoint inhibitors were more successful than investigator’s choice chemotherapy [17, 18]. CheckMate-141 compared nivolumab against standard therapy and showed an OS of 7.7 vs. 5.1 months [18]. KEYNOTE 040 showed that pembrolizumab, as a monotherapy, was superior to chemotherapy and had an OS of 8.4 vs. 6.9 months [17]. Nivolumab and Pembrolizumab have been approved by the FDA for treatment of HNSCC.

Platinum-based chemotherapy has been the primary treatment for NSCLC without driver mutation for many years. Recently, several trials reported that ICIs have a potential role in the treatment of NSCLC. KEYNOTE 024 demonstrated that pembrolizumab monotherapy was superior to platinum-based chemotherapy in patients with PD-L1 expression level above 50% as first-line therapy [19]. Progression-free survival (PFS) was 10.3 vs. 6 months and the ORR was 44.8% vs. 27.8% [19]. KEYNOTE 189 demonstrated that the combination of pembrolizumab with pemetrexed/platinum-based chemotherapy produced better outcomes in first-line therapy when compared to pemetrexed/platinum-based chemotherapy alone [20]. The OS of first-line therapy was 11.3 months and the OS for the PD-1 combination was not yet reached [20]. IMpower 150 studied atezolizumab plus chemotherapeutic regimens, containing a platinum and taxane with bevacizumab, versus the same chemotherapeutic regimen without atezolizumab in NSCLC. The PFS was 8.3 months vs. 6.8 months [21, 22].

It is important to note that studies that have involved combining two ICIs versus combining an ICI with chemotherapy have led to varying results. For advanced melanoma, CheckMate-067 studied ipilimumab versus nivolumab versus a combination of ipilimumab and nivolumab. Ipilimumab and nivolumab alone reported PFS of 2.9–6.9 months whereas the combination of the two had a PFS of 11.5 months [23]. Grade 3–4 adverse events (AEs) occurring in CheckMate-067 ranged from 16.3–55% of patients [23]. While there were many benefits found in the combination of nivolumab with ipilimumab, the high percentage of adverse events led to another clinical study, CheckMate-511. In this study nivolumab and ipilimumab were combined and tested in two different ratios, 3:1 and 1:3. The regimen containing the higher ratio of nivolumab to ipilimumab showed lower AEs, longer PFS (9.9 vs. 8.9 months), but fewer ORRs (45.6% vs. 50.6%) [24].

Immunotherapy combined with chemotherapy or targeted therapy may offer improved clincial outcomes. In addition to the previously mentioned trials KEYNOTE-189 and IMpower150, atezolizumab combined with nab-paclitaxel also provided longer PFS in patients with triple negative breast cancer compared to nab-paclitaxel alone [25]. Furthermore, in patients with renal cell carcinoma, KEYNOTE-426 trial demonstrated that pembrolizumab plus axitinib leaded longer PFS compared to standard sunitinib treatment [26]. From these studies, the combination of immunotherapy with chemotherapy or target therapy not only benefit in longer PFS but also higher objective response rate.

### Immunotherapy associated with biomarkers in tumor microenvironment

Numerous studies have focused on identifying biomarkers that can predict treatment efficacy (Table 3). For example, PD-L1 has proven to be a good predictive biomarker when using pembrolizumab in NSCLC patients. In KEYNOTE 010 trial, patients with PD-L1 levels over 50% had higher ORR, PFS and OS compared to total population [27]. Treatment benefit was further demonstrated in KEYNOTE 024 phase 3 trial, which supported pembrolizumab as first-line therapy for metastatic NSCLC [19]. In KEYNOTE 042 study, the benefit was still observed in patient with tumor proportion score (TPS) greater than 50% compared to those with TPS score 1–49% [28]. However, the correlation between PD-L1 expression level and treatment effect was not observed in other cancer types or in studies with other immunotherapy agents [29–31]. The indications with consideration about PD-L1 expression were listed in Table 4. More recently, Lee et al., reported a novel method to remove the glycosylation of PD-L1. In such cases, de-glycosylation might enhance PD-L1 detection and improve the accuracy of PD-L1 quantification and prediction of PD-1/PD-L1 immune checkpoint blockade therapies [32].

**Table 3:** Predictive biomarkers for treatment efficacy of PD-1/PD-L1 targeting agents

Drug	Sample	Marker	Result	Reference
Pembrolizumab	NSCLC	PD-L1*	↑: longer OS & PFS	Herbst et al., 2016; Reck et al., 2016; Mok et al., 2019
Pembrolizumab	Metastatic colon cancer	MMR*	Deficiency: ORR = 40%, DCR = 90%	Le et al., 2016
Pembrolizumab	11 cancer types	MMR*	Deficiency: ORR = 53%, DCR = 77%	Le et al., 2017
Nivolumab	NSCLC	TMB	↑: longer PFS	Carbone et al., 2017
Nivolumab+Ipilimumab	NSCLC	TMB	↑: longer OS & PFS	Hellmann et al., 2018
Nivolumab	melanoma	ALC	≥ 1000u/L: ↑ prognosis	Nakamura et al. 2016
Nivolumab	melanoma	ANC	< 4000u/L: ↑ prognosis	Nakamura et al. 2016
Ipilimumab	melanoma	NLR + LDH	NLR > 2.2 & ↑LDH: ↓ RR	Bagley et al., 2017
PD-1 targeted therapy	NSCLC	Ki67	↑: positive outcomes	Kamphorst et al., 2017
Pembrolizumab	melanoma	TCR repertoire	↓ diversity: positive clonal responses	Tumeh, et al., 2014
Nivolumab	Metastatic melanoma	*PD-L1* + *GZMA* + *HLA-A*	↑: better clinical outcomes	Hiroyuki et al., 2016
Nivolumab	Metastatic melanoma	TCR repertoire	↓ diversity: ↑ responses	Hiroyuki et al., 2016; Sabrina et al., 2018
PD-1 targeted therapy	melanoma	Ruminococcaceae family	↑ alpha diversity & relative abundance: ↑ responses	Gopalakrishnan et al., 2018
PD-1 targeted therapy	Lung & kidney cancers	Akkermansia muciniphila	↑ relative abundance: ↑ responses	Routy et al., 2018

* PD-L1 and MMR are clinically applicable biomarkers

*NSCLC* Non-small-cell lung carcinoma, *OS* Overall survival, *PFS* Progression free survival, *ORR* Overall response rate, *DCR* Disease control rate, T*MB* Tumor mutation burden, *ALC* Absolute lymphocyte count, *NLR* Neutrophil-to-lymphocyte ratio, *LDH* Lactate dehydrogenase, *RR* Response rate, *TCR* T-cell receptor

**Table 4:** Indications with consideration about biomarkers in advanced cancers

	Pembrolizumab	Nivolumab	Atezolizumab
Breast cancer, triple negative, first-line	–	–	IC ≥ 1%
Cervical cancer, second-line	CPS ≥ 1	–	–
Colorectal cancer, second-line	MSI-high	MSI-high	–
Esophageal cancer, second-line	CPS ≥10	–	–
Gastric cancer, second-line	CPS ≥1	–	–
Head and neck cancer, first-line	CPS ≥1	–	–
Non-small cell lung cancer, first-line	CPS ≥1	–	–
Urothelial carcinoma, first-line	CPS ≥10	–	IC ≥ 5%
Solid tumor, second-line	MSI-high	–	–

*CPS* Combined positive score, *IC* Infiltrating immune cells, *MSI* Microsatellite instability

Mismatch-repair deficiency has also proven to be another practical predictive biomarker for immunotherapy. Le et al. demonstrated that ORR of pembrolizumab in patients with metastatic colon cancer was higher in patients with mismatch-repair deficiency compared to those with mismatch-repair proficiency. In patients with mismatch-repair deficiency, the ORR was 40% and disease control rate was 90%. In contrast, in patients with mismatch repair proficiency, no response could be seen [33]. Overman et al. also reported similar treatment benefit of nivolumab in patients with metastatic colon cancer and mismatch-repair deficiency. In that study, the ORR was 31% and the disease control rate was 69% [34]. Le et al. further demonstrated a treatment benefit of pembrolizumab in solid tumors with mismatch-repair deficiency, including colorectal cancer, endometrial cancer, gastroesophageal cancer and eight other types of cancer. The ORR was 53% and disease control rate was 77% [35]. Taken together, these results offer a strong case for mismatch-repair deficiency as a biomarker in patient selection for immune checkpoint blockade across cancer types. In 2017, U.S. Food and Drug Administration (FDA) approved pembrolizumab for unresectable or metastatic mismatch-repair deficient solid tumors that progressed following prior treatment.

Tumor mutation burden (TMB) has also been widely discussed as a potential predictive biomarker for immunotherapy. In CheckMate 026 study, despite unsuccessful treatment benefit for NSCLC patients with nivolumab or chemotherapy, PFS was significantly longer in high TMB subgroup when separating nivolumab group based on the level of TMB, [36]. In CheckMate 227 trial, nivolumab plus ipilimumab also provided longer PFS and ORR in patients with high TMB compared to those receiving chemotherapy, irrespective of PD-L1 expression level or tumor histology type [37]. Cristescu et al. evaluated hundreds of samples with different cancer types from four trials involving pembrolizumab and found that TMB was correlated with the PFS among groups of pan-tumor, head and neck cancer and melanoma [38]. TMB studies involving liquid biopsies have also demonstrated encouraging results; however, samples from these biopsies are still challenging and inconsistent. Georgiadis et al. used liquid biopsy to test the mismatch-repair deficiency and TMB. Results demonstrated the feasibility of noninvasive screening for mismatch-repair deficiency and TMB in the prediction of PD-1 blockade efficacy [39]. In 2017, Foundation One testing was approved by US FDA for TMB detection.

In spite of its application to aid in patient selection, the assessment of TMB is still plagued by a number of uncertainties. First, TMB has been measured by various methods. Hence, changes in the cut-off definitions as well as alterations in the number of gene panel may affect results. Second, some TMB evidence is obtained from chromosomal structural analyses or mutational status from selected genes [40]. As the reports with LRP1B, KRAS, MSH2 and MSH6 demonstrate, these approaches can only be useful in specific cancer types [41–43]. Third, the difficulties in obtaining sufficient tissue samples as well as good quality of DNA available from biopsy limits the implementation of TMB test. In this respect, standardized evaluation of TMB and better noninvasive sampling methods are needed.

Although PD-L1 expression, mismatch-repair, and TMB are considered potential biomarkers to predict efficacy of various immune therapies, growing evidence suggests other factors like neutrophil to lymphocyte ratio (NLR), lactate dehydrogenase (LDH), and Ki-67 might be valuable markers for prognosis in cancer patients receiving immune therapy. For example, absolute lymphocyte count greater than 1000u/L and absolute neutrophil count less than 4000u/L were reported to be associated with the treatment outcomes in patients with advanced melanoma treated with nivolumab [44]. In addition, high NLR has been shown to be associated with poor response [44]. Hazama et al. reported that NLR < 3.0 correlated with longer survival in cancer patients with peptide vaccine treatment [45]. Recent resources also revealed a critical role for NLR and LDH in the regulation of melanoma treated with ipilimumab [46]. High levels of NLR (greater than 2.2) combined with high serum LDH level are associated with non-response. Importantly, in the lung cancer patients treated with nivolumab, NLR ≥ 5 correlated with poor therapeutic outcome, suggesting NLR is a potential predictive markers in immune therapy [47]. Moreover, NLR was reported as a marker for the outcomes of chemotherapy in advanced cancer [48]. Despite intense investigation and some encouraging results on NLR, the mechanism underlying this correlation remains unclear.

There are still many potential predictive biomarkers for cancer immune therapy. For example, Ki67 is a marker of cell proliferation and T-cell reinvigoration. Kamphorst et al. reported that increase in Ki-67+ PD-1+ CD8 T cells serve as a marker that correlates with positive clinical outcomes for NSCLC patients receiving PD-1–targeted therapies [49]. A particularly important example of how T-cell invigoration can predict response to anti-PD-1 therapy comes from a study in human melanoma. Huang et al. indicated that high Ki67 to tumor burden ratio correlates with a better clinical outcome [50]. In addition, there is considerable evidence for a role of T cell receptor repertoire in cancer immune therapy. In melanoma, Tumeh at al. indicated that low diversity of T cell repertoire in tumor infiltrating lymphocytes associated with positive clinical responses of pembrolizumab [51]. Hiroyuki et al. provided evidence that high expression of PD-1 ligands, granzyme A, and HLA-A correlated with a better clinical outcome with nivolumab. A decreased diversity of T cell repertoire was observed in the tumor tissue of nivolumab responders [52]. Consistent with this were the findings that using peripheral blood T cell receptor repertoire analysis, Sabrina et al. further indicated that low diversity of immune repertoire can be a predictive marker of anti-PD-1 therapy [53]. Recently, most interest has focused on gut microbiome, which is thought to influence the clinical responses of anti-PD-1 immune therapy [54, 55]. Immunoscore (see ‘Current Challenges and future perspectives for PD-1/PD-L1 therapy’) is another area of interest for useful prognostic information about predicting response to treatment. However, the challenge remains in identifying individual immunoprofiles of each patient as well as the consequent choice of optimal therapy to predict drug effect. To date, no single biomarker is considered the gold standard for predictive or clinical use in cancer immunotherapy.

### Adverse events in cancer patients treated with PD-1/PD-L1 blockade

**Fig. 1:**
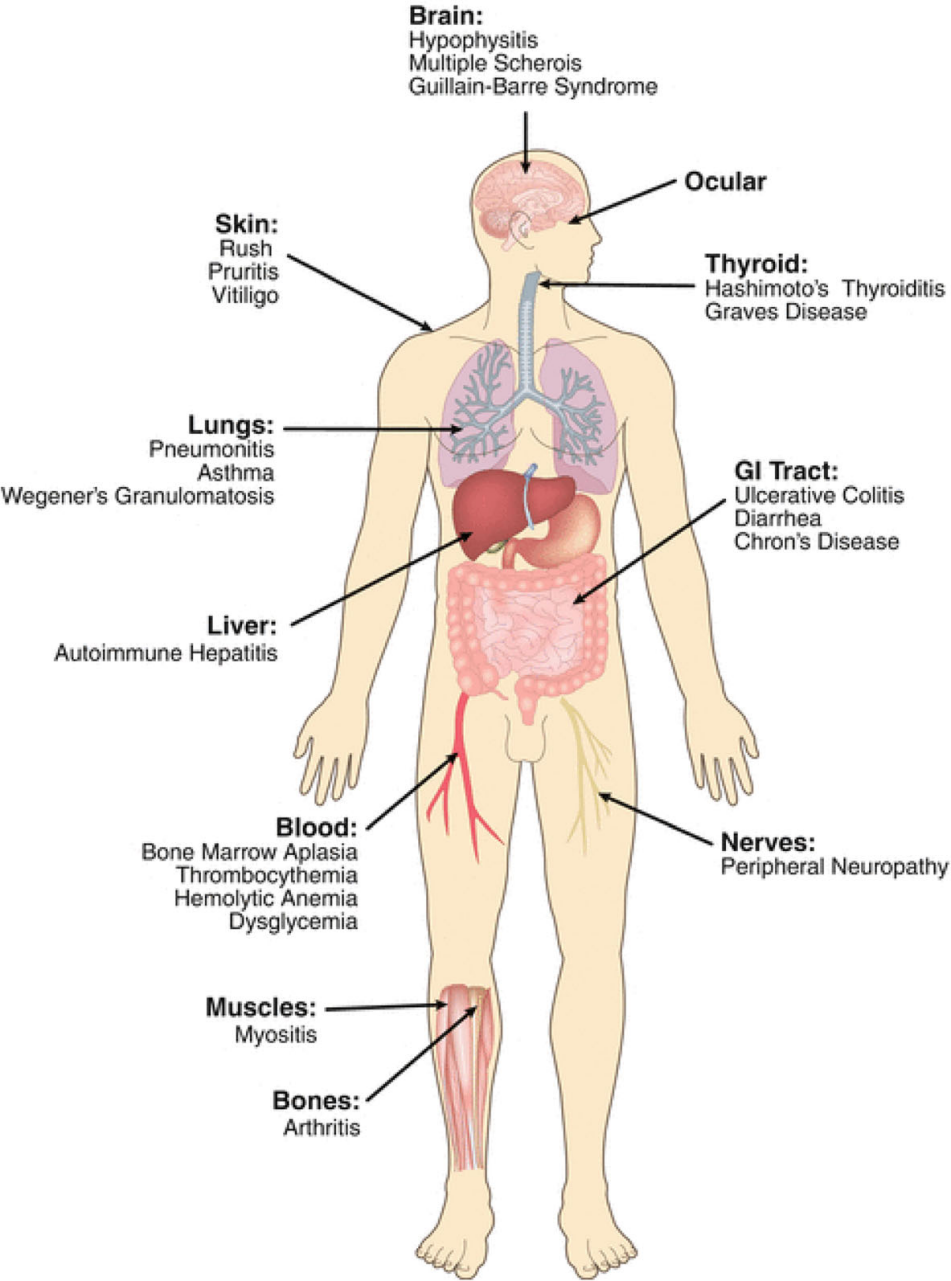
Complete Spectrum of adverse events associated with cancer immunotherapy. Depicted are common immune-related adverse events in patients treated with immune checkpoint blockade (modified from Festino L. and Ascierto P.A. (2018) “Side Effects of Cancer Immunotherapy with Checkpoint Inhibitors.” In: Zitvogel L., Kroemer G. (eds) Oncoimmunology. Springer, Cham)

PD-1/PD-L1 inhibitors are becoming prominent cancer therapies due to their efficacy and their relatively mild adverse events (AEs) compared to chemotherapeutic agents. However, the AEs caused by PD-1/PD-L1 inhibitors are considerable and require further research. Some of the most well documented AEs associated with PD-1/PD-L1 inhibitors fall into several categories: dermatologic, gastrointestinal, hepatic, pulmonary, cardiovascular, and endocrine. Other common AEs include, but are not limited to, fatigue, uveitis and myositis (Fig. 1).

Of the dermatologic AEs associated with PD-1/PD-L1 inhibitors, rash and pruritus are the most predominant [56]. A 2019 meta-analysis by Yang et al. found that patients receiving PD-1/PD-L1 inhibitors have an increased risk of developing pruritus and rash when compared to patients receiving chemotherapy [57]. This same study showed that patients receiving ipilimumab as a monotherapy had an increased risk of developing pruritus compared to patients treated with PD-1/PD-L1 inhibitors. Both of these AEs deeply impact the quality of life for patients. Preventative treatment along with accurate diagnosis of dermatologic AEs can reduce treatment discontinuation and improve overall outcomes.

Gastrointestinal AEs of PD-1/PD-L1 inhibitors include colitis and diarrhea. Symptoms of colitis may include abdominal pain, fever, and abnormal stools. High grade colitis has potentially fatal consequences such as GI tract perforation, ischemia, necrosis, or toxic megacolon [58]. CheckMate 064 reported colitis was the most common treatment-related G3–4 AE and the most common reason for discontinuing treatment [59]. Diarrhea may be a symptom of colitis or a separate AE induced by a checkpoint inhibitor. Regardless, diarrhea must be treated to avoid a hydroelectrolytic imbalance. Symptoms of diarrhea include an increase of number of stools per day that surpass the patient’s baseline [58]. Diarrhea of a G4 AE could include life threatening symptoms such as hemodynamic collapse [58].

Hepatic AEs of PD-1/PD-L1 inhibitors affect a low percentage of patients [60]. However, liver toxicity can be fatal. Patient’s liver function should be monitored closely. Elevated aspartate aminotransferase (AST) and alanine aminotransferase (ALT) are indicators of hepatic AEs. Before treatment with PD-1/PD-L1 inhibitors, a patient’s history of autoimmune disease and/or chronic viral infections should be taken into account. While it is uncommon, hepatitis B/C (HBV/HCV) and/or human immunodeficiency virus (HIV) can be exacerbated by immunotherapy [60, 61]. It is recommended that patients with underlying hepatitis or autoimmune disease be followed by a specialist in their field while receiving PD-1/PD-L1 inhibitors [60, 61].

Pneumonitis can be a fatal AE associated with PD-1/PD-L1 inhibitors [62]. A meta-analysis completed in 2019 found that treatment with PD-1/PD-L1 inhibitors - nivolumab, pembrolizumab, and atezolizumab - increase the risk of pneumonitis [62, 63]. Pembrolizumab was the only PD-1/PD-L1 inhibitor found to have a greater risk of pneumonitis compared to chemotherapeutic agents [62]. Ipilimumab did not demonstrate an increased risk of pneumonitis [62]. The combination of ipilimumab with nivolumab was reported to have more pulmonary AEs than ipilimumab or nivolumab as monotherapies [64].

Myocarditis, an inflammatory AE, is the most common cardiovascular toxicity associated with ICIs [65]. Patients who receive a combination of nivolumab and ipilimumab compared with those who receive nivolumab alone have a higher risk for myocarditis [66, 67]. Presentation of myocarditis could involve elevated serum cardiac biomarkers such as cardiac troponin and creatine kinase-muscle/brain [68]. Myocardial inflammation can also cause shortness of breath and in severe cases lead to cardiogenic shock. The diagnosis of myocarditis requires the use of an MRI scan, PET scan, CT scan, and/or an echocardiogram [65, 66]. In specific cases, an endomyocardial biopsy may be necessary. It is crucial that myocarditis be diagnosed and treated in its early stages as more advanced myocarditis is highly fatal. Patients with ICI induced myocarditis are also seen to have myositis or myasthenia gravis [65, 69]. If a patient presents with myocarditis, it is important to check for other concurrent AEs.

Endocrine AEs include hypothyroidism, hyperthyroidism, and primary adrenal insufficiency. These have been linked to various PD-1/PD-L1 inhibitors. Thyroid disorders are diagnosed by measuring thyroid-stimulating hormone (TSH), thyroxine(T4), triiodothyronine (T3) levels, and thyroid antibodies. Elevated TSH and suppressed T4 indicate hypothyroidism and suppressed TSH and elevated T4 and/or T3 levels indicate hyperthyroidism [70]. Clinical symptoms of thyroid disorders such as fatigue, sensitivity to temperature, constipation, dry skin and fluctuating weight are difficult to differentiate from other diseases [71]. Therefore, measurements of TSH, T4, and T3 levels are crucial for proper diagnosing. A recent Meta-Analysis reported that PD-1/PD-L1 inhibitors have a higher risk of primary thyroid dysfunction when compared to anti-CTLA-4 [72]. Primary adrenal insufficiency is extremely rare but worth noting due to its association with need for life-long treatment and high fatality rates [73]. Low cortisol and high adrenocorticotropic hormone (ACTH) are indicators of primary adrenal insufficiency [70]. Clinical presentations may include asthenia, fever, abdominal pain, vomiting, diarrhea, and weight loss [73].

Myositis and myasthenia gravis are both neuromuscular disorders which can occur with PD-1/PD-L1 inhibitor therapy. Pembrolizumab specifically has been documented to elevate risk of myositis and myasthenia gravis in patients [74]. Symptoms of myositis may include elevated creatine kinase or limb weakness [75]. Interstitial pneumonitis and cardiac toxicity have been found to occur concurrent with myositis [75]. Myasthenia gravis can present with orthopnea, dyspnea, or weakness in facial muscles [75]. While neuromuscular disorders are relatively rare among AEs in patients undergoing immunotherapy, they still require great attention and research so that prompt recognition and treatment can improve outcomes.

Ophthalmologic AEs of PD-1/PD-L1 inhibitors are rare but can deeply impact a patient’s quality of life. Uveitis is generally the most common form of ophthalmologic AEs with symptoms of eye redness, pain, blurred vision, and photophobia [76]. Both ipilimumab and nivolumab as monotherapies have been reported to increase ophthalmologic AEs in patients [76, 77]. Uveitis as an AE is usually minor but, in some cases, can cause blindness and the discontinuation of immunotherapy may be required [78].

### Management of immune-related adverse events in cancer patients treated with PD-1/PD-L1 blockade

**Fig. 2:**
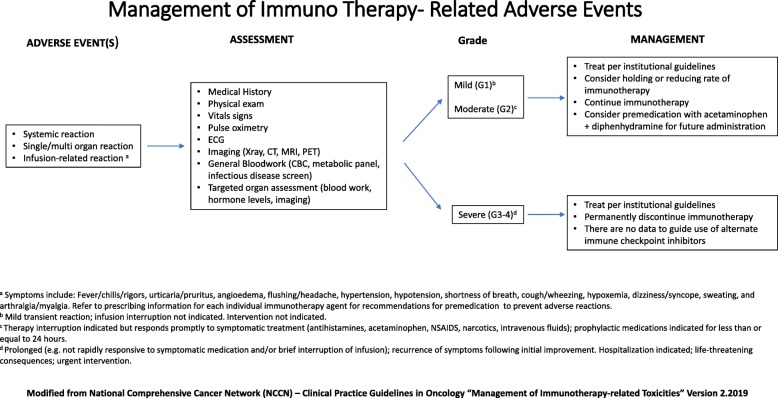
Algorithm of managements of immune-related adverse events

Detailed tracking of AEs secondary to different ICIs will lead to improved patient treatments and outcomes. Some AEs associated with immunotherapy are fatal and other AEs are severe and can deeply diminish patient quality of life. As the study of efficacy regarding PD-1/PD-L1 inhibitors continues, the treatment of immune related AEs (irAEs) must also advance. Detailed algorithms regarding the management of immunotherapy-related toxicities can be found in the National Comprehensive Cancer Network Clinical Practice Guidelines (NCCN Guidelines) (Fig. 2, [79]).

Rash and pruritus are among the most common AEs and usually require a set of general recommendations to keep skin AEs under control. These recommendations include wearing loose clothing, avoiding hot shower/baths, using unscented soaps, applying sunscreen when outside and moisturizing the skin regularly [80]. An itching management should be taught to patients so that a secondary infection does not arise from cuts on the skin. For the treatment of severe rash and pruritus, corticosteroids, antihistamines, antibiotics, or retinoids should be prescribed [81]. Patients with G2–3 dermatologic AEs may require the discontinuation of immunotherapy due to the discomfort and impairment of patient’s quality of life.

Colitis and diarrhea range from relatively mild to fatal AEs. Symptoms of colitis include abdominal pain, fever, and abnormal stools [58, 59]. Higher grade colitis can be potentially fatal [58]. These severe side effects may require an immunosuppressive drug such as infliximab and discontinuation of treatment [58]. Infliximab is an immunosuppressant and works by neutralizing tumor necrosis factor alpha (TNFα) [82]. Infliximab has been used to treat inflammatory colitis such as Crohn’s disease and ulcerative colitis. Lower grade colitis can be treated with corticosteroids, but if the patient does not respond to corticosteroids, infliximab should be given. Treatment for mild to moderate diarrhea includes hydration and a low fiber diet. If diarrhea is severe enough to include life threatening symptoms such as hemodynamic collapse, immediate intravenous fluid resuscitation and discontinuation of treatment is required [58].

Immune-mediated hepatitis is another severe side effect of PD-1/PD-L1 inhibitors. Routine monitoring of liver enzymes allows for prompt diagnosis and treatment of liver toxicities. For G2–4 hepatitis, steroid therapy should be used [83]. In this case, if there is no response to steroid therapy, treatment with infliximab is not recommended as it can further increase hepatotoxicity [83]. A non-respondent steroid patient should undergo a liver biopsy to confirm and clarify hepatitis associated with PD-1/PD-L1 inhibitors. Treatment for a non-respondent steroid patient includes a direct-acting antiviral or ursodiol therapy [83].

Pneumonitis secondary to PD-1/PD-L1 inhibitors can be associated with death. Treatment of pneumonitis most commonly includes corticosteroids, but in some cases involves cyclophosphamide and infliximab [84, 85]. The cessation of treatment is common in G3–4 pneumonitis. Early recognition of pneumonitis is essential for the treatment and recovery of patients.

Myocarditis is not a common AE associated with PD-1/PD-L1 inhibitors but has a high mortality rate. The diagnoses and treatment of myocarditis is extremely important when administering ICIs. Treatment of myocarditis includes use of steroids with other classic heart failure management [67]. Anti-thymocyte globulin, an immunosuppressive treatment, has been reported as an effective drug against myocarditis. Infliximab has been used as a treatment after high-dose steroids fail but has been associated with heart failure in patients with rheumatoid arthritis [86].

Endocrine dysfunctions are common AEs. Hypothyroidism and hyperthyroidism rarely have severe symptoms and can be treated with hormone manipulation [70, 72]. It is advised that thyroid dysfunction, grade 2 or lower, do not require cessation of immunotherapy [70]. Grades 3 and 4 hypothyroidism can be treated with levothyroxine and hyperthyroidism. Grade 3 and 4 hyperthyroidism can be treated with steroids and other forms of therapy to prevent a thyrotoxic storm [70]. Primary adrenal insufficiency should be treated with gluco- and mineralocorticosteriods [70, 72]. Depending on the severity of primary adrenal insufficiency, hormone replacement therapy may be life-long [73].

Myositis and myasthenia gravis are both neuromuscular disorders that can be AEs of PD-1/PD-L1 inhibitors. Myositis treatment includes a combination of steroids, plasmapheresis and intravenous immunoglobulins [87]. Similarly, myasthenia gravis should be treated with corticosteroids and possibly immunosuppressive drugs. In some cases, cholinesterase inhibitors have been given to patients for temporary symptom relief [88].

Uveitis should be taken seriously as it can lead to blindness. Uveitis is treated with systemic or topical steroids [77, 78]. In high grade cases, the complete discontinuation of immunotherapy is required. It is advised to consult both a dermato-oncologist and ophthalmologist [77, 78].

### Current challenges and future perspectives for PD-1/PD-L1 therapy

Immune checkpoint therapies have been clinically observed to induce sustained response in cancer patients; however, most treatment failures are due to primary resistance. In some cases, cancer progresses after the primary response; but this is probably the result of systematic acquired resistance [89, 90]. Such resistance originates from cancer immunoediting comprising of three phases—elimination, equilibrium, and escape—to constrain the immune system and evade detection by the immune system, thereby facilitating tumor growth [91]. An extremely complex tumor microenvironment can explain variability in immune checkpoint therapies. Even in a single patient, metastatic lesions in different areas of the body elicit heterogeneous responses to therapy. Both intrinsic and extrinsic factors of the tumor microenvironment contribute to the development of such resistance. Intrinsic resistance originates from a loss of neoantigens, changes in the antigen presentation mechanism due to dysregulation of major histocompatibility complex (MHC), defective immunosuppressive genes, and immune cell infiltration or function pathway changes [92–96]. Extrinsic factors include the expression of Treg cells, myeloid-derived suppressor cells (MDSCs), M2 macrophages, and other inhibitory immune checkpoint molecules, all of which inhibit antitumor immune responses [89, 97, 98]. Understanding these resistance factors facilitates the development of new strategies for overcoming resistance and provides theoretical support for personalized immunotherapy.

Individual biological differences can explain varied clinical responses to immune checkpoint therapies. Therefore, the ability to predict immune response before administering treatment will be particularly crucial. Researchers have yet to succeed in using specific biomarkers to predict therapeutic effects and treatment-induced toxic responses. Numerous emerging immune checkpoint molecules have been deemed promising targets, but no specific concomitant biomarker has been identified. Therefore, development of novel predictive biomarkers is a pressing matter. The vital criteria to be considered when developing predictive biomarkers are identifying correlations between the biomarker and clinical outcome, low complexity, high reproducibility, low cost, and ease of standardization [99]. Only recent clinical research has looked at specific biomarkers to serve as a basis for application of immune checkpoint inhibitors. Selective CD8+ T-cell infiltration, the distribution of T-cells at tumor invasive margins, and PD-L1 expression were found to be associated with clinical response to anti-PD-1/PD-L1 therapy [51, 100–102]. Studies demonstrate that specific genes involved in chromatin remodeling (i.e., PBRM1, ARID2, and BRD7) can be used as markers for predicting responses. The epithelial mesenchymal transition is highly associated with tumor microenvironment changes including elevated inflammatory signals and enhanced expression of multiple immune checkpoints in lung cancer [103]. Another promising biomarker is a change or defect in the DNA damage response (DDR) pathway, and such DDR variants have also been discovered in numerous tumors [104]. The number and density of tumor-infiltrating lymphocytes can be standardized to form a simple classification system called immunoscore, which might serve as a useful indicator of effectiveness of immune checkpoint therapies with high prognostic value. The immunoscore ranges from I0 (the lowest) to I4 (the highest) and distinguishes tumors (primary or metastatic) according to their degree of immune infiltration, thereby classifying them into two categories—hot and cold. Hot tumors contain high levels of infiltrating T-cells and usually respond favorably to immune checkpoint inhibitors [105, 106]. Cold tumors lack infiltrating T-cells and have low PD-L1 expression, high cell proliferation, and a low mutation burden; moreover, lack of tumor antigenicity and immunogenicity result in no activation of T-cells and thus an unfavorable response to immune checkpoint therapy.

Absence of T-cells at the tumor site also suggests there is no antitumor T-cell response. The CD8+ T-cells at tumor sites play a crucial role in the therapeutic effect of PD-1 inhibitors. Therefore, PD-1 inhibitors are ineffective in the microenvironment of cold tumors. Because hot tumors have highly favorable and multiple inhibitory immune checkpoint molecule expression, the therapeutic strategy for these tumors should involve using multiple brakes on the host immune system to revitalize previously activated T-cells to boost the immune response. Regarding the therapeutic strategy for cold tumors, the microenvironment composition of the tumor should be stimulated through heat before immune checkpoint inhibitors are applied. Literature reports that type I interferon (IFN) and signaling pathway in autophagy are associated with immunogenic cell death (ICD) response. The released danger-associated molecular patterns (DAMP) activated by the immune system’s microenvironment in response to cellular stress and death can promote antigenicity expression [107]. These regulations alter the tumor microenvironment and make it more receptive to immune checkpoint inhibitor therapy. Finally, although application of immune checkpoint inhibitors in cancer treatment show great potential and enormous opportunities, the high price of immunotherapies results in a high cost per life, thus limiting the use of these therapies for suitable patients.

## Conclusion

Recent work reveals a central role of PD-1 signaling pathway in cancer immunotherapy. Although data from clinical trials provides exciting results for PD1/PD-L1 inhibitors in advanced cancer therapy, challenges in clinical use still remain. First, when using PD-1/PD-L1 inhibitor alone without biomarkers selection, the ORR is around 10–25% and time to response is within 2–4 months [17, 18, 30, 31, 108]. For patients with advanced cancer and visceral crisis, these agents do not ensure ability to control tumor in a short time. Second, the costs of PD-1/PD-L1 inhibitors are still expensive. The monthly cost of immunotherapy is around 2–5 times higher compared to the cost of standard targeted therapy [109]. The extremely high cost limits affordability for most patients. Finally, although several factors have been proposed to predict the anti-PD-1 immune therapy, no predictive markers are available for clinical use. To ensure the technical reliability as well as clinical utility of immune therapy for cancer patients, improvements in standardization of predictive biomarker assessments and large-scale randomized trials are warranted.

## Data Availability

Data and materials related to this work are available upon request.

## Abbreviations

AE: Adverse event
ALT: Alanine aminotransferase
AST: Aspartate aminotransferase
CTLA-4: Cytotoxic T-lymphocyte antigen-4
DAMP: Danger-associated molecular patterns
DC: Dendritic cells
DDR: DNA damage response
FDA: Food and Drug Administration
HBV/HCV: Hepatitis B/C
HIV: Human immunodeficiency virus
HNSCC: Head and neck squamous cell carcinoma
ICD: Immunogenic cell death
ICI: Immune-checkpoint inhibitors
IFN: interferon
irAEs: Immune related adverse events
LDH: Lactate dehydrogenase
MDSCs: Myeloid-derived suppressor cells
MHC: Major histocompatibility complex
NLR: Neutrophil to lymphocyte ratio
NSCLC: Non-small cell lung cancer
ORR: Overall response rate
OS: Overall survival
PD-1: Programmed cell death receptor-1
PD-L1: Programmed death-ligand-1
PFS: Progression-free survival
TCR: T cell receptor
TMB: Tumor mutation burden
TPS: Tumor proportion score
