# Correction to: Development and clinical applications of cancer immunotherapy against PD-1 signaling pathway

**DOI:** 10.1186/s12929-019-0601-2

**Published:** 2019-12-23

**Authors:** Grace Wakabayashi, Yu-Ching Lee, Frank Luh, Chun-Nan Kuo, Wei-Chiao Chang, Yun Yen

**Affiliations:** 1 Taipei Medical University, 250 Wu-Hsing Street, Taipei, Taiwan 110; 2 Center for Cancer Transnational Research, Taipei Medical University, 250 Wu-Hsing Street, Taipei, Taiwan 110; 3 Sino-American Cancer Foundation, 668 ArrowGrand Circle, Suite 101, Covina, California 91722 USA; 4 Department of Clinical Pharmacy, School of Pharmacy, Taipei Medical University; Department of Pharmacy, Integrative Therapy Center for Gastroenterologic Cancers, Wan Fang Hospital; Taipei Medical University, 250 Wu-Hsing Street, Taipei, Taiwan 110; 5 PhD Program for Cancer Biology and Drug Discovery, Taipei Medical University, 250 Wu-Hsing Street, Taipei, Taiwan 110

**Correction to: J Biomed Sci**

**https://doi.org/10.1186/s12929-019-0588-8**

In the original publication of this article [1] the name of the fifth author is uncorrect. The correct name of the fifth author should be Wei-Chiao Chang rather than Wei-Chao Chang. The original publication has been corrected.

## References

[1] Wakabayashi (2019). Development and clinical applications of cancer immunotherapy against PD-1 signaling pathway. J Biomed Sci.

